# Folic Acid Homeostasis and Its Pathways Related to Hepatic Oxidation in Adolescent Rats Exposed to Binge Drinking

**DOI:** 10.3390/antiox11020362

**Published:** 2022-02-11

**Authors:** María del Carmen Gallego-Lopez, María Luisa Ojeda, Inés Romero-Herrera, Fátima Nogales, Olimpia Carreras

**Affiliations:** Department of Physiology, Faculty of Pharmacy, Seville University, 41012 Seville, Spain; margallop6@alum.us.es (M.d.C.G.-L.); ineromher@alum.us.es (I.R.-H.); fnogales@us.es (F.N.); olimpia@us.es (O.C.)

**Keywords:** binge drinking, folic acid, oxidative stress, apoptosis, nitrosative stress

## Abstract

Chronic ethanol consumption and liver disease are intimately related to folic acid (FA) homeostasis. Despite the fact that FA decreases lipid oxidation, its mechanisms are not yet well elucidated. Lately, adolescents have been practising binge drinking (BD), consisting of the intake of a high amount of alcohol in a short time; this is a particularly pro-oxidant form of consumption. The aim of this study is to examine, for the first time, FA homeostasis in BD adolescent rats and its antioxidant properties in the liver. We used adolescent rats, including control rats and rats exposed to an intermittent intraperitoneal BD model, supplemented with or without FA. Renal FA reabsorption and renal FA deposits were increased in BD rats; hepatic deposits were decreased, and heart and serum levels remained unaffected. This depletion in the liver was accompanied by higher transaminase levels; an imbalance in the antioxidant endogenous enzymatic system; lipid and protein oxidation; a decrease in glutathione (GSH) levels; hyper-homocysteinemia (HHcy); an increase in NADPH oxidase (NOX) 1 and NOX4 enzymes; an increase in caspase 9 and 3; and a decrease in the anti-apoptotic metallopeptidase inhibitor 1. Furthermore, BD exposure increased the expression of uncoupled endothelial nitric oxide synthase (eNOS) by increasing reactive nitrogen species generation and the nitration of tyrosine proteins. When FA was administered, hepatic FA levels returned to normal levels; transaminase and lipid and protein oxidation also decreased. Its antioxidant activity was due, in part, to the modulation of superoxide dismutase activity, GSH synthesis and NOX1, NOX4 and caspase expression. FA reduced HHcy and increased the expression of coupled eNOS by increasing tetrahydrobiopterin expression, avoiding nitrosative stress. In conclusion, FA homeostasis and its antioxidant properties are affected in BD adolescent rats, making it clear that this vitamin plays an important role in the oxidative, nitrosative and apoptotic hepatic damage generated by acute ethanol exposure. For this, FA supplementation becomes a potential BD therapy for adolescents, preventing future acute alcohol-related harms.

## 1. Introduction

The relationship between folic acid (FA), chronic ethanol consumption (Chr-EtOH) and alcoholic liver disease (ALD) is well recognized in clinical and experimental research. Chr-EtOH suppresses key biological processes directly relevant to FA and folate metabolism, such as folate intestinal absorption, liver uptake and storage, urinary excretion, and folate enzymes synthesis and methylation; it also leads to lower folate intake [1]. Therefore, currently, FA is supplied to Chr-EtOH patients in order to prevent ALD, as this is the main vitamin deficiency from which they suffer [2].

The mechanisms of Chr-EtOH injury are multifactorial, involving several pathways, most of them related to its oxidative metabolism, which takes place mainly in the liver. The principal product of this metabolism is acetaldehyde, which leads to changes in the reduced and oxidized nicotinamide adenine dinucleotide (NADH-NAD^+^) ratio, increasing mitochondrial oxidation. One of these EtOH metabolizer enzymes is CYP2E1, which produces acetaldehyde but also reactive oxygen species (ROS) directly, increasing oxidative stress (OS) [3]. Biomolecular oxidative damage is considered one of the main mechanisms related to ALD.

Synthetic FA, together with natural folates, is a member of the B9 vitamin family and is essential for amino acid metabolism. FA is taken up from the diet and bio-transformed mainly in the liver by the enzyme dihidrofolate reductase (DHFR). It functions as a coenzyme in reactions of one-carbon transfer, needed for the biosynthesis of several essential molecules [4]. Adequate intake of FA is vital for metabolism, cellular homeostasis and DNA synthesis [5]. In addition, in the last meta-analysis displayed, it is suggested that FA supplementation improves the antioxidant defense system by increasing serum concentrations of glutathione (GSH) and decreasing lipid oxidation [6]. Despite the fact that different authors have described this property, all studies approach the issue from a one-sided point of view. For instance, “in vitro” studies describe FA as a novel treatment to combat hepatocellular carcinoma by modulating ROS sinker proteins and maintaining mitochondrial redox homeostasis [7]. Other “in vitro” studies suggest a direct action of FA on OS generation as a ROS scavenger, acting as a reducing agent [8]; however, this action is not clear “in vivo” [9,10]. Most authors relate the antioxidant properties of FA to its ability to lower the pro-oxidant homocysteine (Hcy). Hyper-homocysteinemia (HHcy) increases hepatic and renal nicotinamide adenine dinucleotide phosphate (NADPH) oxidase (NOX) activity, which in turn leads to high amounts of ROS production, increasing lipid peroxidation, and it also affects the mRNA levels of the mitochondrial NOX4, the mitochondrial Complex II and calcium levels, contributing to apoptosis [11,12,13]. In the kidneys, Hcy also increases oxidation by affecting the activities of the pro-oxidant xanthine oxidase and the antioxidant superoxide dismutase (SOD) [12]. FA metabolism is deeply related to the Hcy metabolism through the methionine cycle. Therefore, in the liver, FA supplementation inhibits NOX-ROS production, decreasing OS, by decreasing Hcy [11]. Recently, Ju et al. suggested that FA, by inducing the mitochondrial enzyme methylene tetrahydrofolate dehydrogenase 2 (MTHFD_2_), could modulate NOX4 and redox homeostasis together to modulate apoptosis in colorectal cancer [14]. However, all these authors point to other FA antioxidant effects not related to Hcy remethylation. FA stimulates the activity of the enzyme γ-glutamylcysteine synthetase (γ-GCS), increasing the transsulfuration pathway, and the synthesis of GSH. GSH is an endogenous antioxidant that is also necessary for the correct function of the endogenous antioxidant enzyme glutathione peroxidase (GPx) [15]. Finally, different authors studying endothelial cells point to FA as an important protector from hypoxia by decreasing both ROS and apoptosis, linked to NOX4 pathways by the induction of nitric oxide (NO) production. This action is attributed to the fact that FA increases endothelial NO synthase (eNOS) in its coupled form, which is the antioxidant form [16]. This mechanism is linked to the upregulation of the enzyme DHFR in response to FA administration, which enhances tetrahydrobiopterin (BH4), promoting eNOS recoupling [17,18,19]. In this regard, there is not a well-elucidated overview of all these mechanisms in the same study.

Binge drinking (BD) is an acute way of consuming EtOH, defined by the National Institute on Alcohol Abuse and Alcoholism (NIAAA) as a pattern of drinking alcohol that brings blood alcohol concentration (BAC) to 0.08% or higher (≥0.08 g/dL) during a short period of time [20]. Before and during the COVID-19 pandemic, this strong method of EtOH consumption has been the most widespread among teenagers [21,22], being a public health concern since adolescence is a stage that is especially vulnerable to the toxic effects of EtOH. Acute EtOH consumption leads to different biological effects than Chr-EtOH, since in its metabolism, it produces higher amounts of ROS by increasing CYP2E1 activity [23]. BD exposure is an especially potent pro-oxidant that could produce different changes in FA homeostasis, compromising the oxidative balance even more. Nevertheless, there are few studies related to acute EtOH consumption and FA homeostasis. In human volunteers, serum FA levels fell and returned rapidly to normal values after acute alcohol exposure [24]. The mechanisms for these quick phenomena remain unexplained. In this context, our research group found that in adolescent rats, 12 h after BD exposure, serum FA levels were unaltered and peripheral oxidation took place. When these animals were supplemented with FA, serum FA levels increased and lipid and DNA oxidation significantly decreased [25]. However, plasma levels of metabolites are not a simple reflection of changes in liver levels of the same metabolites [26]; therefore, FA tissue deposits should be analyzed. In this study, for the first time, FA tissue levels are measured together with FA intake and FA in serum and urine in order to increase knowledge about FA homeostasis in adolescent rats exposed to BD. Moreover, to understand the possible repercussion of this on the oxidative disruption provoked by BD exposure, different oxidative pathways will be analyzed at the same time “in vivo”, also in FA-supplemented adolescent rats. We will obtain a general overview of FA involvement in ROS and reactive nitrogen species (RNS) over-production, OS/nitrosative stress (NS) balance and biomolecular damage which could induce apoptosis during BD exposure.

## 2. Materials and Methods

### 2.1. Experimental Design

A total of 24 Wistar male adolescent rats (Centre of Production and Animal Experimentation, Vice-Rector’s Office for Scientific Research, University of Seville) were used to execute this experiment. These animals were received at 21 days old and housed in groups of 2 rats per cage for 1 week with free access to food and drink to acclimatize them to housing and handling conditions. The animals were kept in the animal house of the Faculty of Pharmacy at an automatically controlled temperature (22–23 °C) and a 12 h light–dark cycle (07:00–19:00). At 28 days of age, when Wistar rats initiate the adolescence age, the animals were randomly divided into 4 experimental groups (*n* = 6): control group (C): rats were fed with commercial feed (2 ppm FA) and water ad libitum and, 3 days a week, they received an intraperitoneal (i.p.) injection of physiological saline solution (PSS); binge drinking group (BD): rats were fed with commercial feed (2 ppm FA) and water ad libitum and, 3 days a week, they received an i.p. injection of ethanol (3 g/kg); control folic acid group (CF): rats were fed with commercial feed supplemented with FA (8 ppm FA) and water ad libitum and, 3 days a week, they received an i.p. injection of PSS; and binge drinking folic acid group (BDF): rats were fed with commercial feed supplemented with FA (8 ppm FA) and water ad libitum and, 3 days a week, they received an i.p. injection of ethanol (3 g/kg). The animals remained under this treatment for 3 weeks (47 days old), at which point they were humanely sacrificed. Throughout the complete experimental period (28 to 47 days old), the rats suffered the typical-age alterations characteristic of adolescence, including those of the puberty phase; according to the review of Spear [27], there is an abundance of evidence that confirms that the animal model used in this experiment can be extrapolated to human adolescents, such as behaving like an adolescent or initiating alcohol and the consumption of other drugs. Animal care procedures and experimental protocols were in accordance with EU regulations (Council Directive 86/609/EEC, 24 November 1986) and were approved by the Ethics Committee of the University of Seville (CEEA-US2019-4).

### 2.2. Diet Used

The diet used during the experimental protocol was a standard pellet diet (2014 Teklad Global 14% Protein Rodent Maintenance Diet, Harlan Laboratories, Barcelona, Spain) that covered all their nutritional and energy requirements, with an FA content of 2 ppm. Supplemented groups (CF and BDF) received an FA-supplemented diet prepared by adding a fine FA powder (ACOFARMA, Barcelona, Spain) at 8 ppm to the crushed standard pellet diet. Later on, supplemented biscuits were made by reconstituting the mixture with water and were dried at room temperature. Fresh batches of diet were made each week.

### 2.3. Binge Drinking Treatment

EtOH exposed groups (BD and BDF) received the alcohol treatment called “binge drinking” by the administration of EtOH (3 g/kg) in PSS at 20% (*v*/*v*) via i.p. during 3 consecutive days per week for 3 weeks [28]. No i.p. injections were given during the remaining four days of each week. This i.p. forced BD method has been chosen in order to avoid digestive interferences with FA oral supplementation and to analyze the direct effect of this drug on the body. On the other hand, control groups (C and CF) were administered an equivalent volume of PSS i.p. over the same period. The injections were given starting at 7:00 pm when the dark cycle began.

### 2.4. Nutritional Control

Each day during the three weeks of treatment, the food intake (g/day) was determined by measuring the difference between the feed placed and the feed remaining the next day using an analytical balance. FA intake (µg/day) was calculated from the data of the food intake, taking into account that the feed for the supplemented animals was prepared with a concentration of 8 ppm of FA and the base diet contained 2 ppm. At the same time, to monitor the development of the animals during the experiment, they were weighed every day using an analytical balance. The measurements were always taken at the same time, between 9:00–10:00 a.m., to avoid differences due to the circadian rhythm.

### 2.5. Samples

Once the experimental period was over, the rats fasted for 12 h in individual metabolic cages, where urine samples were collected. Following that, 24 h after their last treatment with EtOH or PSS, the animals were weighed and anesthetized with an i.p. injection of 28% *w*/*v* urethane (Sigma-Aldrich, Madrid, Spain) (0.5 mL/100 g of body weight). Then, blood was obtained by intracardiac puncture through the thorax and placed in tubes. The clot was allowed to retract for 30 min at room temperature, and then it was centrifuged at 1300× *g* for 15 min to prepare the serum. Finally, when the blood was removed, the abdomen of each rat was opened by a midline incision, and the whole livers were extracted, debrided of adipose and connective tissue in ice-cold saline and weighed in order to calculate the liver somatic index (LSI% = liver weight/total rat weight). The kidneys and heart were also removed. All samples were frozen in liquid nitrogen and stored at −80 °C for future biochemical determinations.

### 2.6. Serum Measurements

The serum levels of Hcy and the transaminases alanine aminotransferase (ALT) and aspartate aminotransferase (AST) were measured with an automated analyzer (Technicon RA-1000, Bayer Diagnostics, Leverkusen, Germany). Metallopeptidase inhibitor 1 (TIMP-1) was analyzed by the Luminex xMAP (Millipore, Darmstadt, Germany), a system that combines three basic forms of xMAP technology.

### 2.7. Folic Acid Homeostasis

Serum, urine, liver, kidneys and heart FA levels were analyzed by ELISA using the “Folic Acid ELISA Kit” from CELL BIOLABS, INC. (MET-5068). This analysis was carried out with the reagents supplied by the manufacturer and according to their specifications in a 96-well plate. The Folic Acid ELISA Kit is a competitive enzyme immunoassay developed for rapid detection and quantification of FA. The FA standards and the samples are added to a FA conjugate preabsorbed microplate. After a brief incubation, an anti-folic acid polyclonal antibody is added, followed by an HRP conjugated secondary antibody. The amount of FA in the samples is quantified by comparing its absorbance at 450 nm with the FA standard curve. Regarding the preparation of samples, the tissues (liver, kidneys, heart) were homogenized (1:10 *w*/*v*) in ice-cold PBS using a Potter homogenizer (Pobel 245432, Madrid, Spain). The homogenates were incubated at 4 °C for 20 min, and then they were centrifugated at 12,000× *g* for 20 min. The urine samples were diluted (1:5) in Assay Diluent.

### 2.8. Activity of Antioxidant Enzymes and Oxidative Stress Markers in the Liver

Liver tissue samples were homogenized (1:4 *w*/*v*) using a Potter homogenizer (Pobel 245432, Madrid, Spain) in a sucrose buffer (15 mM Tris/HCl, pH 7.4, 250 mM sucrose, 1 mM EDTA and 1 mM dithiothreitol) in an ice bath. Then, the homogenates were centrifuged at 3000 r.p.m for 20 min at 4 °C and kept at −80 °C until the time of analysis. The activity of SOD (U/mg protein) was determined by the Fridovich method, which is based on the ability of SOD to inhibit the reduction of cytochrome c induced by the xanthine-xanthine oxidase system; it is measured by the absorbance increase at 550 nm for 3 min, due to the reduction of cytochrome c by adding xanthine oxidase [29]. Catalase (CAT) activity (U/mg protein) was determined using H_2_O_2_ as substrate by the assay of Beers and Sizer, where the disappearance of H_2_O_2_ was followed spectrophotometrically at 240 nm for 3 min [30]. The GPx activity (mU/mg) was determined using the method described by Lawrence and Burk [31]. In this assay, the oxidized glutathione (GSSG) formed by the action of GPx is coupled to the reaction that catalyzes the glutathione reductase (GR) enzyme, measuring the absorbance decrease at 340 nm for 3 min due to the oxidation of NADPH. The GR activity (mU/mg protein) was analyzed according to the spectrophotometric method described by Worthington and Rosemeye using GSH as substrate, where the absorbance decrease at 340 nm during 3 min due to the oxidation of NADPH was measured [32]. In order to measure the OS markers in the liver, the amount of lipid peroxidation was evaluated by the quantification of malondialdehyde (MDA) (mol/mg protein), the end-product of oxidative degradation of lipids, using a colorimetric reaction with thiobarbituric acid (TBA) at 535 nm as described by Draper and Hadley [33]. The protein oxidation was measured by the detection of carbonyl groups (CG) (nmol/mg protein) at 366 nm by the method described by Reznick and Packer, where the reaction of 2,4-dinitrophenylhydrazine (DNPH) with CG takes place [34].

### 2.9. GSH Hepatic Levels

To measure GSH hepatic deposits, liver homogenates were prepared in 50 mmol/L potassium phosphate buffer (pH 7.5) containing 1 mmol/L EDTA and 1 mg/mL bovine serum albumin with the Potter homogenizer (Pobel 245432, Madrid, Spain). Then, they were centrifuged at 3000 r.p.m for 20 min using the resulting supernatant fraction. Total GSH levels in the samples were determined by a colorimetric method using a commercial kit (Bioxytech GSH/GSSG-412, Oxis Research, Beverly Hills, CA, USA).

### 2.10. Nitric Oxide Hepatic Levels

Livers were homogenized in ice-cold PBS (1:10) using a Potter homogenizer (Pobel 245432, Madrid, Spain). Homogenates were incubated at 4 °C for 20 min and centrifuged for 20 min at 12,000× *g*. Before the assay, the homogenates were filtered with 10,000 MWCO polysulfone filters (Vivaspin 500 SARTORIUS, Stonehouse, UK). The NO levels in the liver were analyzed by the Nitric Oxide Detection Kit (ADI-917-010) from Enzo Life Sciences Inc. (Barcelona, Spain). This kit quantitatively determines nitrate and nitrite concentrations using the enzyme nitrate reductase, which converts nitrate into nitrite, followed by the colorimetric detection at 540 nm of nitrite as a colored azo dye product from the Griess reaction. The NO levels in a system are measured by the determination of both nitrate and nitrite concentrations in the sample.

### 2.11. Immunoblotting Analysis in the Liver

The livers of adolescent rats were homogenized (1:10 *w*/*v*) in 50 mM phosphate buffer (K_2_HPO_4_ 50 mM, KH_2_PO_4_ 50 mM, EDTA 0.01 mM, protease inhibitor 1:10 (Complete Protease Inhibitor Cocktail Tablets, ROCHE, Madrid, Spain) using the Potter homogenizer (Pobel 245432, Madrid, Spain). Then, homogenates were centrifuged at 2000 r.p.m at 4 °C for 10 min, and the final supernatant was aliquoted and frozen at −80 °C until analysis.

The expression of the hepatic proteins NOX1, NOX4, eNOS, DHFR, nitrotyrosine, caspase-9, caspase-3 and β-actin (as load control) in the previously described homogenates was carried out using the protein immunodetection technique or Western blot. After determining the protein content of the homogenates, the appropriate volumes (100 µg/lane) were mixed with an equal volume of sample buffer (Sample Buffer, Laemmli 2X Concentrate SIGMA) plus 5% of 2-mercaptoethanol (BIORAD, Madrid, Spain). Proteins were separated by SDS-polyacrylamide gel electrophoresis (PAGE) using acrylamide gels (6% for eNOS and 12% for the rest of proteins). Samples were heated at 95 °C for 5 min before electrophoresis to denature proteins, except for dimer and monomer eNOS. In this case, in order to separate the dimer and the monomer of eNOS, samples were not heated. They did not contain 2-mercaptoethanol, and the temperature of the gel was maintained below 15 °C during electrophoresis (low-temperature SDS-PAGE). The proteins were then transferred onto a nitrocellulose membrane using a blot system for 120 min. Membranes were blocked next for 1 h at room temperature with blocking buffer composed of TBS (10 mM Tris-HCl, 100 mM NaCl, pH 7.5) and 5% milk powder (BIORAD, Madrid, Spain). For immunodetection of the proteins, membranes were incubated overnight at 4 °C with specific primary antibodies (mouse monoclonal IgG, Santa Cruz Biotechnology, Heidelberg, Germany) in a dilution of 1:1000. Monoclonal mouse anti β-actin (IgG1A5441, Sigma-Aldrich, Madrid, Spain) was used to detect β-actin as a loading control with a dilution of 1:10000. The next day, after 54 min washes with TBST (TBS + 0.1% Tween 20, PanReac Applichem, Barcelona, Spain), probed membranes were incubated with the secondary antibody (Goat Anti-Mouse IgG (H + L)-HRP Conjugate, BIORAD, Madrid, Spain) in a dilution of 1:2500 for all proteins and 1:8000 for β-actin. The membranes were incubated for 1 min with the commercial developer solution Luminol ECL reagent (GE Healthcare and Lumigen Inc., Buckinghamshire, UK) and analyzed with the Amersham Imager 600 (GE Healthcare, Buckinghamshire, UK). Then, the quantification of the blots was performed by densitometry with the ImageJ program. The results were expressed as percent arbitrary relative units, referring to values in control animals which were defined as 100%.

### 2.12. Ratios of Measurements

The hepatic ratios of GR/GPx, GR/GSH and cleaved caspase-3/caspase-3 were calculated. The results were expressed as percentages with regard to the values in control animals, defined as GR and cleaved caspase-3 100%.

### 2.13. Statistical Analysis

The results are expressed as the mean ± standard error of the mean (SEM), where “*n*” is the number of samples used. The means shown were obtained from 12 data (*n* = 12) for biochemical determinations or from 6 data (*n* = 6) for proteins expression. To compare the different variable objects of our study in the four experimental groups, the data were analyzed using the analysis of variance (ANOVA) in the program GraphPad InStat 3 (San Diego, CA, USA). Subsequently, the Tukey–Kramer test was used to determine the significant differences between the means, considering statistically significant differences with values of *p* < 0.05.

## 3. Results

### 3.1. Nutritional and Hepatic Parameters

Intermittent i.p. BD exposure to adolescent rats did not alter food intake, body weight gain, relative liver weight or hepatic protein content. However, transaminase levels, specifically of AST and ALT, were significantly increased (F(3, 20) = 7.7, *p* < 0.001; (F(3, 20) = 4.8, *p* < 0.05 for AST and ALT, respectively) (Table 1). FA supplementation did not affect any of those parameters, but in BD-exposed rats it decreased AST (F(3,20) = 3.5, *p* < 0.05) and especially ALT (F(3,20) = 5.1, *p* < 0.01) levels.

### 3.2. Folic Acid Homeostasis

**Figure 1:**
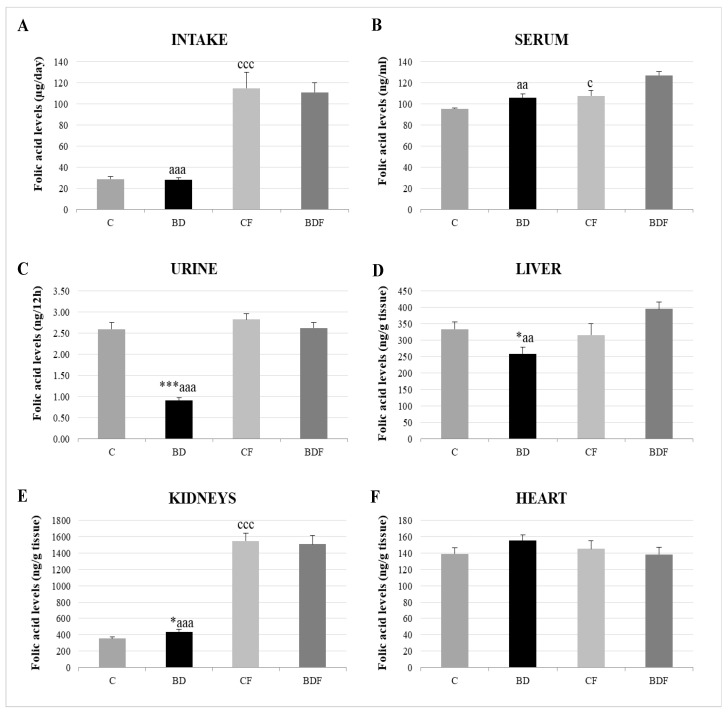
Folic acid homeostasis in the rats’ bodies. Intake levels (**A**), serum levels (**B**), urine levels (**C**) and tissue deposit levels in the liver (**D**), kidneys (**E**) and heart (**F**). The results are expressed as mean ± SEM and analysed by a multifactorial one-way ANOVA followed by Tukey’s test. The number of animals in each group is 6 and *n* = 12. SEM: standard error of the mean. Groups: C: control group, BD: binge drinking group, CF: control folic group, BDF: binge drinking folic group. Signification: C vs. BD: * *p* < 0.05, *** *p* < 0.001; C vs. CF: c *p* < 0.05, ccc *p* < 0.001; BD vs. BDF: aa *p* < 0.01, aaa *p* < 0.001.

With respect to FA homeostasis (Figure 1), BD exposition did not affect FA intake or serum FA levels, but it did lead to significantly low renal FA excretion (F(3.20) = 13.6, *p* < 0.001). This rise in FA renal reabsorption increased renal FA deposits (F(3.20) = 3.2, *p* < 0.05); however, hepatic FA levels were decreased (F(3.20) = 3.5, *p* < 0.05) and heart levels were unaffected. After FA supplementation, FA intake significantly increased in control and BD animals, together with higher levels in serum, the liver and, mainly, in the kidneys.

### 3.3. Oxidative Hepatic Balance

**Figure 2:**
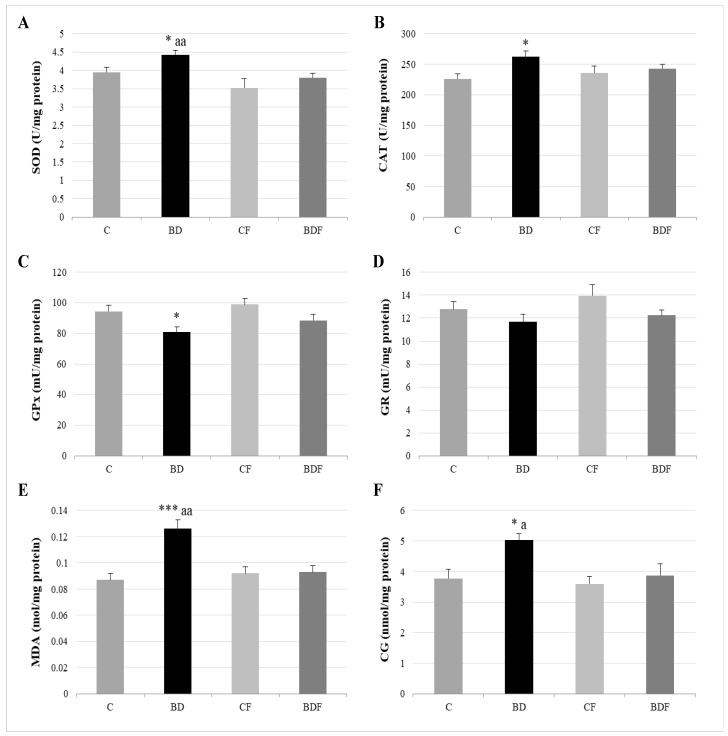
Hepatic oxidative balance: activity of antioxidant enzymes and oxidative stress markers. Superoxide dismutase (SOD) (**A**), catalase (CAT) (**B**), glutathione peroxidase (GPx) (**C**), glutathione reductase (GR) (**D**), lipid oxidation expressed by the levels of malondialdehyde (MDA) (**E**) and protein oxidation expressed by the levels of carbonyl groups (CG) (**F**). The results are expressed as mean ± SEM and analysed by a multifactorial one-way ANOVA followed by Tukey’s test. The number of animals in each group is 6 and *n* = 12. SEM: standard error of the mean. Groups: C: control group, BD: binge drinking group, CF: control folic group, BDF: binge drinking folic group. Signification: C vs. BD: * *p* < 0.05, *** *p* < 0.001; BD vs. BDF: a *p* < 0.05; aa *p* < 0.01.

**Figure 3:**
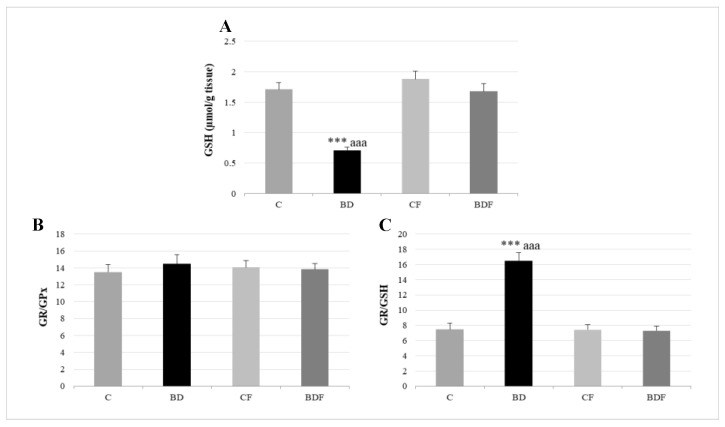
Hepatic GSH levels and their relationship to GPx and GR enzymes. GSH (**A**), GR/GPx ratio (**B**) and GR/GSH ratio (**C**) in the liver. The results are expressed as mean ± SEM and analysed by a multifactorial one-way ANOVA followed by Tukey’s test. The number of animals in each group is 6 and *n* = 12. SEM: standard error of the mean. Groups: C: control group, BD: binge drinking group, CF: control folic group, BDF: binge drinking folic group. Signification: C vs. BD: *** *p* < 0.001; BD vs. BDF: aaa *p* < 0.001.

In the liver, BD exposure significantly increased SOD and CAT activities (F(3.20) = 3.2 and 3.9, *p* < 0.05 to SOD and CAT, respectively) and decreased GPx activity (F(3.20) = 3.8, *p* < 0.05) leading to protein (F(3.20) = 4.3, *p* < 0.05) and lipid oxidation (F(3.20) = 10.29, *p* < 0.001). FA supplementation to BD animals reduced SOD activity (F(3.20) = 6.1, *p* < 0.01) and decreased lipid (F(3.20) = 5.2, *p* < 0.01) and protein oxidation (F(3.20) = 4.1, *p* < 0.05) (Figure 2). BD exposition also significantly decreased GSH hepatic levels (F(3.20) = 9.9, *p* < 0.001); however the ratio of GR/GPx was unaffected, and the GR/GSH ratio was significantly increased (F(3.20) = 12.5, *p* < 0.001). FA supplementation to BD rats significantly increased hepatic GSH levels (F(3.20) = 9.6, *p* < 0.001), leading to control levels of the ratios GR/GPx and GR/GSH (Figure 3).

### 3.4. Serum Homocysteine and NOX Hepatic Expression

**Figure 4:**
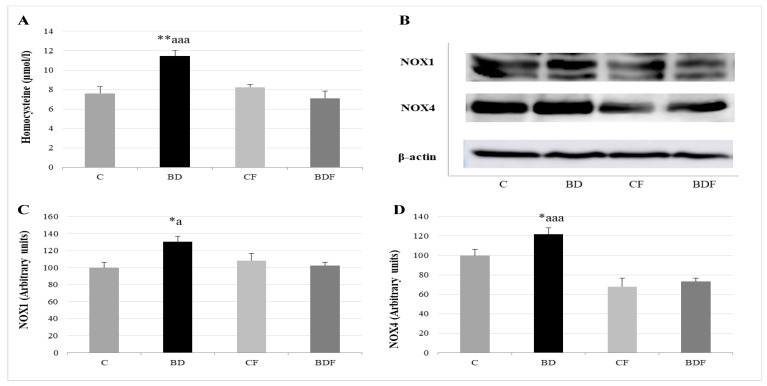
Study of serum homocysteine levels and their relationship with hepatic pro-oxidant enzymes NOX1 and NOX4. Homocysteine levels in serum (**A**). Representative Western blot for the expressions of NOX1, NOX4 and β-actin (as load control) (**B**). Expression of NOX in the liver of adolescent rats (expressed as percent arbitrary relative units, referring to values in control animals which were defined as 100%): NOX1 (**C**) and NOX4 (**D**). The results are expressed as mean ± SEM and analysed by a multifactorial one-way ANOVA followed by Tukey’s test. The number of animals in each group is 6 and *n* = 12 for homocysteine analysis or *n* = 6 for NOX1 and NOX4 expression determinations. NOX: NADPH oxidases; SEM: standard error of the mean. Groups: C: control group, BD: binge drinking group, CF: control folic group, BDF: binge drinking folic group. Signification: C vs. BD: * *p* < 0.05, ** *p* < 0.01; BD vs. BDF: a *p* < 0.05, aaa *p* < 0.001.

After BD consumption, serum Hcy levels significantly rise (F(3.20) = 6.4, *p* < 0.01), together with NOX1 and NOX4 liver expression (F(3.20) = 4.7 and 4.3, *p* < 0.05 to NOX1 and NOX4, respectively). FA supplementation to control animals did not affect any of these parameters; however, FA supplementation to BD rats decreased Hcy levels (F(3.20) = 10.52, *p* < 0.001) and NOX1 and NOX4 expression (F(3.20) 4.3, *p* < 0.05 to NOX1, F(3.20) = 9.65, *p* < 0.001 to NOX4) (Figure 4).

### 3.5. Hepatic eNOS Expression

**Figure 5:**
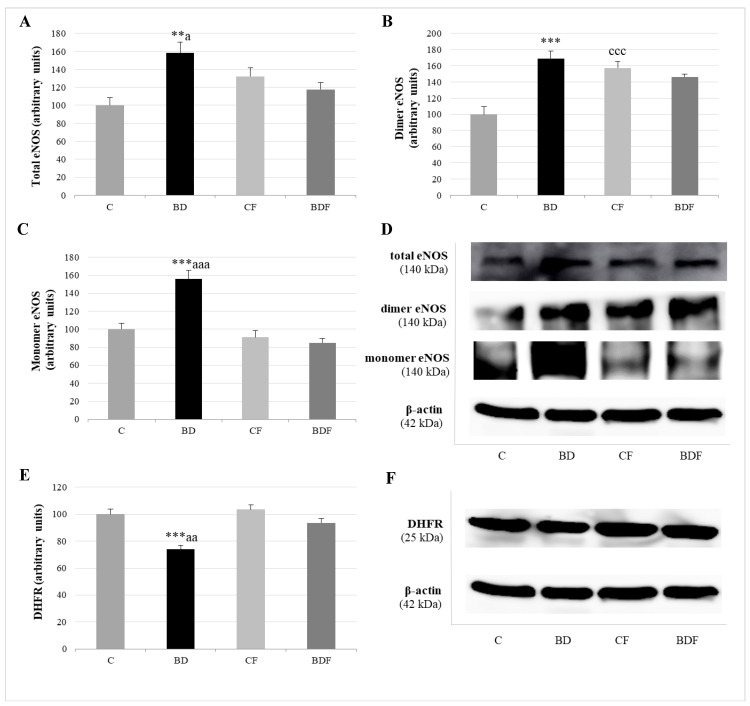
Role of DHFR enzyme in the expression of coupled and uncoupled eNOS in the liver of adolescent rats. Expression of eNOS (expressed as percent arbitrary relative units, referring to values in control animals which were defined as 100%): total eNOS (**A**), dimer-coupled eNOS (producer of NO) (**B**) and monomer-uncoupled eNOS (producer of O_2_^•−^) (**C**). Representative Western blot for the expressions of total, dimer and monomer eNOS and β-actin (as load control) (**D**). Expression of DHFR in the liver of adolescent rats (expressed as percent arbitrary relative units, referring to values in control animals which were defined as 100%) (**E**). Representative Western blot for the expressions of DHFR and β-actin (as load control) (**F**). The results are expressed as mean ± SEM and analysed by a multifactorial one-way ANOVA followed by Tukey’s test. The number of animals in each group is 6 and *n* = 6 for eNOS and DHFR expression determinations. eNOS: endothelial nitric oxide synthase. NO: nitric oxide. O_2_^•−^: superoxide anion. DHFR: dihidrofolate reductase. SEM: standard error of the mean. Groups: C: control group, BD: binge drinking group, CF: control folic group, BDF: binge drinking folic group. Signification: C vs. BD: ** *p* < 0.01, *** *p* < 0.001; C vs. CF: ccc *p* < 0.001; BD vs. BDF: a *p* < 0.05, aa *p* < 0.01, aaa *p* < 0.001.

Figure 5 analyses total eNOS (eNOSt), eNOS dimer or couple eNOS, which generates NO, and eNOS monomer or uncoupled eNOS with a pro-oxidative function. The expression of the enzyme DHFR, which increased BH4, promoting eNOS recoupling, was also measured in the liver. BD exposure increased eNOS in its total (F(3.20) = 6.0, *p* < 0.01), dimer (F(3.20) = 8.8, *p* < 0.001) and monomer (F(3.20) = 7.7, *p* < 0.001) forms, but decreased DHFR expression (F(3.20) = 7.8, *p* < 0.001). FA administration to control rats significantly increased eNOS dimer form (F(3.20) = 7.3, *p* < 0.001). FA supplemented to BD rats decreased eNOSt (F(3.20) = 4.2, *p* < 0.05), mainly by reducing eNOS monomer form (F(3.20) = 9.8, *p* < 0.001), since it increased DHFR expression (F(3.20) = 5.8, *p* < 0.01).

### 3.6. Hepatic Nitrosative Stress

**Figure 6:**
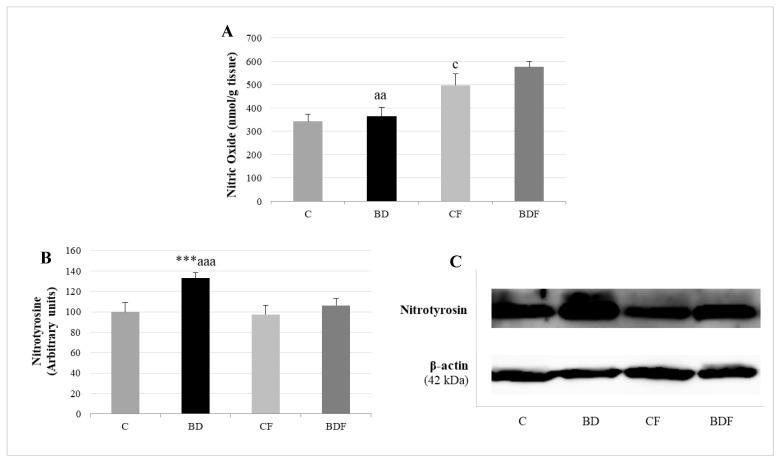
Nitrosative stress analysis by determining NO levels and nitrotyrosine expression in the liver of adolescent rats. Nitric oxide levels (**A**). Expression of nitrotyrosine (expressed as percent arbitrary relative units, referring to values in control animals which were defined as 100%) (**B**). Representative Western blot for the expressions of nitrotyrosine and β-actin (as load control) (**C**). The results are expressed as mean ± SEM and analysed by a multifactorial one-way ANOVA followed by Tukey’s test. The number of animals in each group is 6 and *n* = 12 for NO analysis or *n* = 6 for nitrotyrosine expression determinations. SEM: standard error of the mean. Groups: C: control group, BD: binge drinking group, CF: control folic group, BDF: binge drinking folic group. Signification: C vs. BD: *** *p* < 0.001; C vs. CF: c *p* < 0.05; BD vs. BDF: aa *p* < 0.01, aaa *p* < 0.001.

BD exposure did not increase NO hepatic levels, but it did increase the expression of proteins with nitrotyrosine (F(3.20) = 9.5, *p* < 0.001). FA supplemented to control rats increased the amount of NO without affecting nitrotyrosine expression (F(3.20) = 4.2, *p* < 0.05). FA supplemented to BD rats increased NO hepatic levels and significantly decreased nitrotyrosine expression in hepatic proteins (F(3.20) = 5.8, *p* < 0.01 for NO levels, F(3.20) = 7.7, *p* < 0.001 for nitrotyrosine expression), avoiding nitrosative damage (Figure 6).

### 3.7. Liver Apoptosis

**Figure 7:**
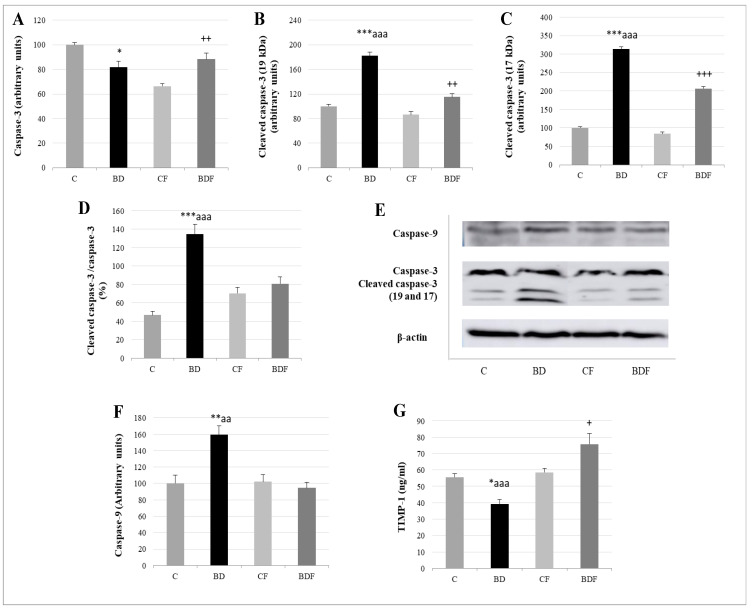
Apoptosis analysis in the liver of adolescent rats. Caspase-3 (**A**), cleaved caspase-3 (19kDa: (**B**) and 17 kDa: (**C**)) expression (expressed as percent arbitrary relative units, referring to values in control animals which were defined as 100%). Ratio of activated caspase-3 (**D**). Representative Western blot for the expressions of caspase-3, cleaved caspase-3, caspase-9 and β-actin (as load control) (**E**). Caspase-9 expression (expressed as percent arbitrary relative units, referring to values in control animals which were defined as 100%) (**F**). Serum TIMP-1 levels (ng/mL) (**G**). The results are expressed as mean ± SEM and analysed by a multifactorial one-way ANOVA followed by Tukey’s test. The number of animals in each group is 6 and *n* = 12 for TIMP-1 analysis or *n* = 6 for caspase expression determinations. SEM: standard error of the mean. Groups: C: control group, BD: binge drinking group, CF: control folic group, BDF: binge drinking folic group. Signification: C vs. BD: * *p* < 0.05, ** *p* < 0.01, *** *p* < 0.001; BD vs. BDF: aa *p* < 0.01, aaa *p* < 0.001; BDF vs. CF: + *p* < 0.05, ++ *p* < 0.01, +++ *p* < 0.001.

Relative to apoptotic studies (Figure 7), BD-exposed animals presented lower caspase-3 levels but higher cleaved caspase-3 expression, leading to a high ratio of cleaved caspase-3/caspase-3 (F(3.20) = 11.7, *p* < 0.001). When FA was supplied, this ratio significantly decreased (F(3.20) = 7.9, *p* > 0.001); however, these animals (BDF) present higher caspase-3 and cleaved caspase-3 than their control counterparts (CF) without differences in the final ratio. BD animals also present higher cleaved caspase-9 expression (F(3.20) = 6.2, *p* < 0.01) and lower serum levels of the anti-apoptotic TIMP-1. When FA was supplemented, BDF rats presented lower caspase-9 expression (F(3.20) = 9.96, *p* < 0.01) and higher levels of TIMP-1 (F(3.20) = 10.5, *p* < 0.001), even higher than CF rats (F(3.20) = 4.9, *p* < 0.05).

## 4. Discussion

### 4.1. Hepatic Function and FA Homeostasis

From a nutritional and morphological point of view, neither i.p. BD exposure nor FA supplementation provoked changes in this study. However, BD rats presented high ALT and AST serum values; this increase in transaminases indicates that hepatic damage is taking place [35]. The same results of transaminases and hepatic damage are described in clinical trials in adolescents exposed to intensive alcohol consumption [36] and in experimental BD-exposed animals [37,38]. When FA is supplemented, these values decrease, particularly ALT values. Despite the fact that AST is located in many tissues, ALT is not usually found outside the liver [39]. Therefore, FA supplementation during BD exposure seems to act specifically in the liver by improving its function, indicating that during BD, liver FA deposits could be compromised.

As was mentioned previously, BD consumption during adolescence does not affect either FA dietary intake or FA serum levels. Nevertheless, renal reabsorption of FA is significantly increased, indicating that the body is making an effort not to excrete it in urine, increasing its retention. Therefore, renal FA deposits are increased but, as was expected, not hepatic deposits. Probably, hepatic FA is consumed in this tissue in order to avoid oxidative EtOH damage, as also happens with other exogenous antioxidants such as selenium [40]. It is also known that superoxide anion (O_2_^•−^) generated by EtOH degrades FA and its main active metabolite 5-methyltetrahydrofolate, being an important catabolic mechanism that contributes to the increased requirement for hepatic folate [41]. Heart FA deposits are not affected by BD exposure.

According to what was previously stated, FA supplementation during BD exposure increases FA intake and serum levels without affecting its renal reabsorption, leading to an increase in liver deposits that does not appear in cardiac muscle. This implies that more FA is necessary in the liver of BD adolescent rats, where it probably plays an important antioxidant function. This theory is reliable since, in CF animals, this increase does not take place. Supplemented animals (CF and BDF groups) present a significant increase in renal FA deposits, pointing to kidneys as an important reservoir of body FA and a tissue to analyze when FA supplementation therapies are used [42].

### 4.2. Hepatic Antioxidant Endogenous Enzymatic Defense System

In this study, BD was found to lead to an important imbalance in the antioxidant enzymatic defense system, increasing SOD and CAT activities and decreasing GPx activity. These changes in the antioxidant system make the detoxification of ROS generated by EtOH difficult, contributing to the generation of lipid and protein oxidation. FA supplementation avoids this biomolecular oxidation in part by decreasing SOD activity. SOD catalyzes the dismutation of the ROS O_2_^•−^ into molecular oxygen and hydrogen peroxide (H_2_O_2_). The action of FA decreasing SOD activity could be due to its supposed effects as a scavenger directly decreasing O_2_^•−^ concentration [8]; by inducing other antioxidant mechanisms that indirectly decrease O_2_^•−^ generation, such as decreasing NOX activity [11,14]; or by decreasing Hcy levels, which stimulates SOD [12]. Moreover, this decrease of lipid and protein oxidation is attributable not only to the decrease of SOD but also to different potential FA-antioxidant actions.

### 4.3. Hepatic GSH Levels

Confirming previous data [43], hepatic antioxidant GSH deposits in the liver of BD-exposed adolescent rats are reduced. BD decreases the hepatic levels of GSH by various mechanisms: EtOH increases the consumption of GSH in cells to neutralize ROS; EtOH inhibits the γ-GCS enzyme, which is necessary for the last step in the transsulfuration pathway to generate GSH; and it decreases the methionine cycle function essential for GSH synthesis [25]. GSH is a natural antioxidant which acts as a scavenger, directly quenching reactive hydroxyl free radicals, other oxygen-centered free radicals, and radical centers on DNA. However, it is also the co-substrate of GPx, permitting the reduction of peroxides and producing glutathione disulfide (GSSG). In turn, GSSG is reduced to GSH by the enzyme GR using NADPH-reducing equivalents. GSH has another important role; it is conjugated with electrophilic endogenous compounds and drugs through the activation of glutathione-S-transferases (GSTs), facilitating toxic elimination [15]. After BD exposure, GSH deposits are decreased, but the antioxidant pair GPx/GR acts harmoniously, since this ratio is not affected, despite GPx being reduced. This means that the decreased activity of GPx is not due to a low amount of GSH, since GR activity is balanced with GPx activity. Its decrease is mainly due to the fact that selenium, a mineral that forms part of its catalytic center, is significantly decreased in BD adolescent rats [40]. Moreover, when hepatic GR activity is relativized to GSH deposit levels, the ratio is deeply increased, so the amount of GSH re-synthesized by this route and destined to maintain GPx antioxidant activity is the main pathway to generating it. Therefore, the methionine cycle and transsulfuration pathways are critical points in GSH generation during BD exposure. In this context, the low remaining hepatic GSH levels are compromised for other important uses such as hepatic detoxification.

When FA is supplied to BD rats, FA hepatic depletion is avoided, hepatic GSH deposit levels increase, the antioxidant pair GPx/GR balances and the GR/GSH ratio is similar to that in control animals. These data confirm that hepatic GSH levels during BD in rats are deeply related to FA deposits and it is regenerated mainly by the transsulfuration pathway, which is reestablished after FA supplementation. Different mechanisms previously described support these results. FA controls the synthesis of GSH by upregulating the γ-GCS enzyme in the transsulfuration pathway; it also modulates the methionine cycle necessary for GSH synthesis and has an important role in the regulation of mitochondrial isocitrate dehydrogenase-2 (ICDH-2), which is responsible for providing the NADPH necessary for regenerating GSSG back to GSH via mitochondrial GR [7].

### 4.4. Hcy Levels in Serum and Hepatic Pro-Oxidant Expression of NOX1 and NOX4

It has been demonstrated for the first time that BD exposure during adolescence, as in chronic alcoholic patients, leads to HHcy [44]. Alterations in hepatic Hcy metabolism are reflected by altered levels of circulating plasma Hcy concentrations, and this is correlated with diverse pathologies, such as vascular dysfunction, neurodegenerative disorders and of course, hepatic injury, including those generated by EtOH consumption [45].

Hcy, a non-essential amino acid, is synthesized by the transmethylation of the essential amino acid methionine, and remethylated to it again by the methionine cycle in all tissues. However, its detoxification through the transsulfuration pathways occurs mainly in the liver [46], with this tissue playing a central role in Hcy metabolism. Therefore, deteriorated liver function has emerged as a major factor in the development of HHcy. This situation probably takes place in BD adolescent rats since they have high transaminase levels [47]. In ALD, high Hcy is related to the accumulation of toxic metabolites, steatosis, apoptosis and OS [48]. Therefore, oxidation plays an important role among Hcy’s proposed toxicity mechanisms [49,50].

It is probable that BD-EtOH exposure contributes to increased serum Hcy levels by altering, as in Chr-EtOH exposition, the methionine cycle and transsulfuration pathway by increasing S-adenosylhomocysteine (SAH) and decreasing hepatic S-adenosylmethionine (SAM), as well as decreasing γ-GCS, leading to lower GSH synthesis. These effects of EtOH are attributed to the ROS generated, and they are exacerbated by a lack of hepatic FA that serves as a cofactor in these pathways [44,51]. Both situations have been detected in BD adolescent rats. According to the previously described situations, FA supplementation in BD rats avoids HHcy, and all its potential damage on adolescent rats, by restoring these pathways and acting as an antioxidant.

As mentioned in the introduction section, Hcy is considered a pro-oxidant agent that compromises the oxidative balance by different mechanisms [46,49,50]. Among its proposed mechanisms is the increase of multicomponent NOX enzyme complexes, which are one of the major producers of endogenous ROS in different tissues [11,12,13,45]. In this context, NOX plays a central role in liver fibrogenesis. NOX1 is expressed in hepatic stellate cells and is related to liver inflammation and fibrosis. NOX4 is expressed in hepatocytes, stellate cells and endothelial cells, and is also related to hepatic fibrosis, even in those of EtOH etiology [45,52,53,54].

It is known that BD as a potent pro-oxidant stimulates NOX1 expression in the liver of adolescent rats exposed to BD [43]. In this study, BD exposure increases NOX1, and especially NOX4, expression, affecting lipid and protein oxidation and apoptosis generation, as will be discussed later. In this context, the pivotal role of NOX4 in chronic alcohol-induced liver injury is described since it is related to mitochondrial activity. Sun et al. proved that Chr-EtOH exposure induced NOX4 expression in the mitochondrial fraction from hepatic cells [55]. Therefore, inhibition of NOX4 activity protects against alcohol-induced fat accumulation and activation of intrinsic apoptosis via improving mitochondrial function. It is known that dysfunctional mitochondria release cytochrome C to the cytosol after alcohol exposure, leading to apoptosis activation. These authors confirm that this release was due in part to the ROS generated by NOX4.

Once more, when FA is administered to BD rats, HHcy is avoided, and NOX1 and NOX4 hepatic expressions are decreased, contributing to reduced lipid and protein oxidation and apoptosis. This is probably due to FA contributing to restoring Hcy remethylation to methionine or to catabolizing it through the transsulfuration pathway, increasing GSH hepatic levels. It is also interesting to bear in mind that FA decreases NOX4 expression in particular. It could perhaps be acting directly on hepatic NOX4 expression, which is also backed by the results obtained by Ju et al. in colon cells, by inducing the mitochondrial MTHFD_2_ enzyme [14]. It is also recognized that FA inhibits NOX activity in hepatic cells by reducing the mRNA levels of several essential NOX subunits [56]. Moreover, some authors concluded that FA could decrease NOX4 activity in endothelial cells by the induction of eNOS [16]. In fact, a new NOX4/DHFR/eNOS uncoupling pathway related to FA in the diet has recently been described in aortic cells [57].

### 4.5. eNOS, NO and NS

The eNOS enzyme is a dimer that depends on the cofactor BH4 to produce NO. When either cofactor bioavailability is limited or when OS appears, the eNOS dimer is destabilized and uncoupled, resulting in the production of O_2_^•−^ rather than NO. In this context, BD increases total hepatic eNOS expression, but either forms coupled or dimer (NO generator) and uncoupled or monomer (O_2_^•−^ generator). BH4 generation depends on the enzyme DHFR, which transforms dihydrobiopterin (BH2) to BH4 [19]. DHFR is decreased in the liver of BD adolescent rats. The enzyme DHFR depends on FA levels, connecting with the lower FA hepatic deposits found in these animals [58]. BH4 depletion is the main cause of the eNOS uncoupling process [59], even in adolescents exposed to high amounts of EtOH [60]. It is transformed to its inactive form BH2 in pro-oxidant ambients, such as that which is generated in the liver during BD exposure. Therefore, the hepatic OS generated by BD exposure could also be contributing to lower eNOS recoupling. Moreover, the eNOS form depends on the pair GSH/GSSG; when this ratio decreases, S-glutathionylation appears, and the enzyme changes into its uncoupled form [61]. Since BD decreases GSH deposits, the ratio is downregulated, and eNOS S-glutathionylation could be affecting eNOS function, leading to O_2_^•−^ generation.

The presence of NO and ROS in the liver of BD adolescent rats involves the formation of nitrosylating intermediates, mainly peroxinitrite (ONOO^−^), which contributes to the formation of S-nitrosylated proteins (protein-SNOs), indicating that NS is also established [62,63]. In the liver, the balance between coupled and uncoupled eNOS is very important for NO homeostasis and ROS production in hepatic sinusoidal endothelial cells. Its imbalance has been observed in cirrhotic livers, being implicated in inflammation and fibrosis [64]. Therefore, this is another piece of biomolecular data that implies that BD exposure during adolescence has an important impact on liver function.

FA therapy by restoring DHFR protein hepatic expression leads to recoupled eNOS expression in BD rats. It increases NO production and decreases protein-SNOs in the liver, avoiding NS. Different mechanisms are related to FA and eNOS activity in endothelial cells, including greater bioavailability of BH4 through stabilization of BH4 and/or recycling from BH2, mainly by upregulating the activity of DHFR in the biopterin recycling pathway, direct interaction with eNOS, and directly decreasing ROS generation [65]. Moreover, since FA increases GSH synthesis, eNOS S-glutathionylation could be avoided, contributing to stabilizing its coupled form.

### 4.6. Apoptosis

Caspases are a family of protease enzymes essential in programmed cell death via apoptosis. Caspase-9 is an initiator caspase related to the intrinsic apoptotic pathway, which appears when mitochondrial cytochrome c is released into the cytosol after cell stress. This cytochrome c recruits caspase-9, then caspase-9 activates other executioner caspases, such as caspase-3. Finally, caspase-3 leads to the degradation of cellular components [66]. BD-exposed rats present with significantly high caspase-9 expression in the liver, which implies that mitochondria are under a stressor condition. This is in agreement with the NOX4 upregulation found in the liver, which generates ROS specifically in this organelle [55]. The ratio of cleaved caspase-3/caspase-3 is also significantly increased in these animals, confirming that apoptosis is established. Moreover, TIMP-1 serum levels are reduced in BD rats. Apart from modulating extracellular matrix remodeling by controlling the activity of matrix metalloproteinases (MMPs), this protein also acts by signaling molecules with cytokine-like activity, having anti-apoptotic functions [67]. It is clear that i.p. BD exposure during adolescence leads to important biomolecular changes in the liver by generating OS and NS. It mainly affects NOX4 expression and specifically damages mitochondrial function, which contributes to increased intrinsic apoptotic pathways.

After FA supplementation, caspase-9 expression is decreased according to the lower levels of NOX4 found in the liver of these animals. This indicates that FA protects liver mitochondria from EtOH damage. Moreover, the ratio of cleaved caspase-3/caspase-3 is also reduced, indicating that apoptosis is decreased when FA hepatic levels are balanced, and that this vitamin plays an important role during BD liver damage. FA is known for its anti-apoptotic properties in different types of cells [13,68,69,70,71]. These authors attribute this action to different mechanisms: through decreasing NOX activity-ROS generation- mitochondrial complex II; decreasing OS-, preventing telomeric DNA oxidation and attrition; regulating the expression of apoptosis-related genes (decreased caspase-3 and upregulated BCL2/BAX ratio); modulating microRNA-34a, associated with Bcl-2 signaling; or decreasing caspase-9 activity. In this context, FA supplementation also increases TIMP-1 serum levels. Among the mechanisms attributed to TIMP-1 in apoptosis is its relationship to the anti-apoptotic protein of the Bcl-family, Bcl-2. This protein increases TIMP-1 expression, and TIMP-1 also stabilizes Bcl-2/BAX, resulting in an upregulation of Bcl-2 and a decrease of the pro-apoptotic Bcl-2-associated X (Bax) protein, avoiding cytochrome-c release by mitochondria [67]. This correlation between FA supplementation and TIMP-1 increase in BD-exposed rats reinforces the idea that FA plays an important role in avoiding mitochondrial damage and apoptosis.

## 5. Conclusions

**Figure 8:**
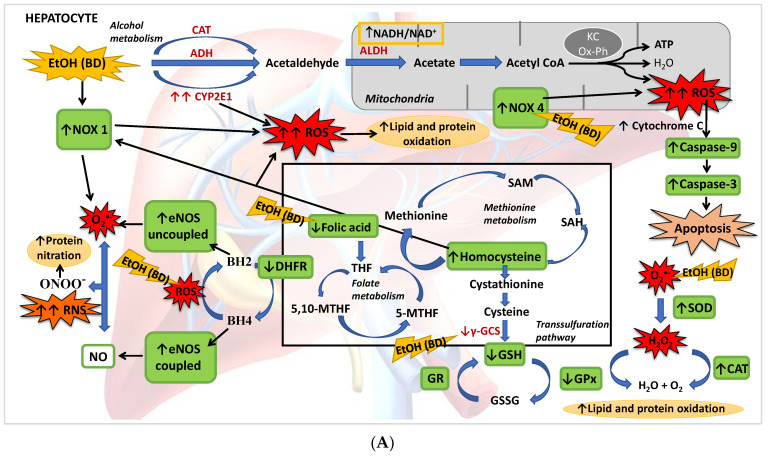

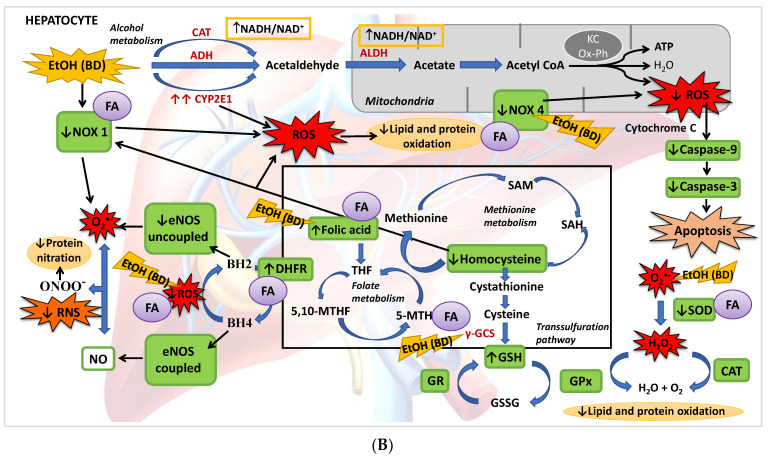
(**A**) BD in adolescence increases the hepatic production of ROS and RNS, causing oxidative and nitrosative stress leading to apoptosis. BD stimulates the pro-oxidant enzymes CYP2E1 and NOX1, increasing cytosolic ROS generation, and NOX4 activity, increasing ROS mitochondrial generation and cytochrome C expulsion, contributing to apoptosis. BD affects the antioxidant enzymatic defense system, increasing SOD and CAT activities and decreasing GPx activity, but lipid and protein oxidation appears. BD affects the methionine cycle and the transsulfuration pathway, leading to depletion in GSH levels and HHcy. BD reduces the expression of DHFR, decreasing the cofactor BH4 and increasing the uncoupled form of eNOS, producing RNS and protein nitration. BD also leads to a hepatic depletion of FA, which could be implicated in all these actions. (**B**) FA antioxidant pathways against hepatic oxidation induced by BD in adolescence. FA supplementation to BD rats manages to reduce the oxidation of lipids and proteins, as well as protein nitration and apoptosis. It decreases the expression of the pro-oxidant enzymes NOX1 and NOX4, avoiding ROS generation and apoptosis. FA restores the balance of the antioxidant enzymatic defense system. Due to its participation in the methionine cycle and the transsulfuration pathway, it increases the levels of the antioxidant GSH and decreases the pro-oxidant Hcy. FA also increases the expression of DHFR and BH4 viability, increasing the stability of coupled eNOS, avoiding RNS generation and nitrosative damage.

The present study described for the first time that BD exposure during adolescence in male rats leads to a depletion of FA hepatic deposits together with a low GSH synthesis and higher Hcy serum levels. This implies that, apart from its oxidative damage by generating ROS and affecting the antioxidant enzyme system, the methionine cycle and transsulfuration pathway are affected after BD exposure, in part by the depletion of FA, increasing hepatic damage. It has also been found that BD leads to NS by increasing eNOs in its uncoupled form, either by its direct pro-oxidant action or by decreasing the GSH/GSSG ratio. Finally, this OS leads to apoptosis mainly by increasing NOX4 activity and mitochondrial damage (Figure 8A). When FA depletion is avoided using a supplemented FA diet, most of these situations are avoided, showing that this vitamin plays a pivotal role in BD hepatic damage. Collectively, we add to the literature new data to strengthen the notion that FA is a vitamin that confers a novel role to combat ROS/RNS insults after BD exposure in adolescents since FA decreases lipid and protein hepatic oxidation, NS and apoptosis by different simultaneous mechanisms. Some of these mechanisms depend on its own antioxidant properties, such as the decrease of NOX4 expression, and others are related to its action on the methionine cycle and transsulfuration pathway, which contributes to increased hepatic GSH (antioxidant) levels and decreased serum Hcy (pro-oxidant) levels. Furthermore, FA contributes to recoupling the eNOS enzyme by restoring DHFR protein abundance and reducing ROS production, avoiding NS. It is important to remark that in vivo treatment with FA completely attenuates NOX4 expression after BD exposure, decreasing mitochondrial damage and apoptosis (Figure 8B). Therefore, FA seems to be a suitable supplement to avoid hepatic damage after BD exposure in adolescents.

## Figures and Tables

**Table 1:** Nutritional and hepatic parameters.

Parameters	C	BD	CF	BDF
Body weight increase (g/day)	5.72 ± 0.17	5.29 ± 0.14	5.80 ± 0.21	5.20 ± 0.53
Food intake (g/day)	14.29 ± 1.31	13.84 ± 1.09	14.35 ± 1.85	13.95 ± 1.04
LSI (%)	3.48 ± 0.17	3.43 ± 0.067	3.65 ± 0.054	3.65 ± 0.12
Liver protein content (mg/g liver wet tissue)	8.95 ± 0.33	9.53 ± 0.50	9.37 ± 0.50	9.19 ± 0.89
Serum AST (U/L)	118 ± 13	210 ± 8 *** a	112 ± 13	168 ± 13 +
Serum ALT (U/L)	29.4 ± 1.4	38.8 ± 2.2 * aa	27.6 ± 1.7	29.12 ± 2.2

The results are expressed as mean ± SEM and analysed by a multifactorial one-way ANOVA followed by Tukey’s test. The number of animals in each group is 6 and *n* = 12. LSI: liver somatic index. AST: aspartate aminotransferase. ALT: alanine aminotransferase. SEM: standard error of the mean. Groups: C: control group, BD: binge drinking group, CF: control folic group, BDF: binge drinking folic group. Signification: C vs. BD: * *p* < 0.05, *** *p* < 0.001; BD vs. BDF: a: *p* < 0.05, aa *p* < 0.01; BDF vs. CF: + *p* < 0.05.

## Data Availability

Data is contained within this article.
